# Internal Jugular and Subclavian Vein Thrombosis in a Case of Ovarian Cancer

**DOI:** 10.1155/2017/5748402

**Published:** 2017-01-17

**Authors:** Hiroto Moriwaki, Nana Hayama, Shouko Morozumi, Mika Nakano, Akari Nakayama, Yoshiomi Takahata, Yuusuke Sakaguchi, Natsuki Inoue, Toshiki Kubota, Akiko Takenoya, Yoshiko Ishii, Haruka Okubo, Souta Yamaguchi, Tsuyoshi Ono, Toshiaki Oharaseki, Mamoru Yoshikawa

**Affiliations:** ^1^Department of Otorhinolaryngology, Toho University Ohashi Medical Center, Tokyo, Japan; ^2^Division of Cardiovascular Medicine, Toho University Ohashi Medical Center, Tokyo, Japan; ^3^Department of Pathology, Toho University Ohashi Medical Center, Tokyo, Japan

## Abstract

Central venous catheter insertion and cancer represent some of the important predisposing factors for deep venous thrombosis (DVT). DVT usually develops in the lower extremities, and venous thrombosis of the upper extremities is uncommon. Early diagnosis and treatment of deep venous thrombosis are of importance, because it is a precursor of complications such as pulmonary embolism and postthrombotic syndrome. A 47-year-old woman visited our department with painful swelling on the left side of her neck. Initial examination revealed swelling of the region extending from the left neck to the shoulder without any redness of the overlying skin. Laboratory tests showed a white blood cell count of 5,800/mm^3^ and an elevated serum C-reactive protein of 4.51 mg/dL. Computed tomography (CT) of the neck revealed a vascular filling defect in the left internal jugular vein to left subclavian vein region, with the venous lumina completely occluded with dense soft tissue. On the basis of the findings, we made the diagnosis of thrombosis of the left internal jugular and left subclavian veins. The patient was begun on treatment with oral rivaroxaban, but the left shoulder pain worsened. She was then admitted to the hospital and treated by balloon thrombectomy and thrombolytic therapy, which led to improvement of the left subclavian venous occlusion. Histopathologic examination of the removed thrombus revealed adenocarcinoma cells, indicating hematogenous dissemination of malignant cells.

## 1. Introduction

Cancer is the second cause of upper extremity deep venous thrombosis (UEDVT), only surpassed by central venous catheter (CVA) insertion [1]. Other predisposing factors include surgery, intravenous drug use, and infection; UEDVT has also been described as an acute complication of pharyngeal bacterial infection, known as Lemierre's syndrome. The percentage of patients with UEDVT among admitted patients with a CVC is relatively high [2], so that the disorder can be diagnosed without difficulty in patients having a CVC and presenting with neck swelling and/or pain. It is of importance, however, to note that occasional cases are asymptomatic. Since it is difficult to immediately entertain the suspicion of UEDVT in patients without a CVC, interpretation of the symptoms and meticulous history taking are important to avoid overlooking the diagnosis. UEDVT, like lower-extremity venous thrombosis (LEDVT), can lead to complications such as pulmonary embolism (PE), sepsis with septic emboli, intracranial propagation of the thrombus with cerebral edema, and postthrombotic syndrome. PE reportedly occurs in 36% of patients with UEDVT [2]; therefore, the importance of prompt and appropriate treatment of UEDVT cannot be overemphasized.

Existence of a relationship between malignant disease and thromboembolic disorders has been known for a number of decades, having first been reported by Trousseau in 1865 [3]. Tumor cells express tissue factors that activate the clotting cascade, tumor procoagulants, fibrinolytic proteins and receptors for these factors, promoting interactions between the tumor cells, platelets and endothelial cells via cytokines, tumor antigens, and their immune complexes to facilitate thrombogenesis [4]. Phlebothrombosis is a known complication of cancer [5], and many published studies have reported cases in which the detection of venous thrombosis led to the diagnosis of cancer, even in patients without a history of malignancy [6]. Thus, in patients diagnosed as having DVT, systematic medical workup is necessary to identify any occult cancer. In cancer patients, death from DVT is second in frequency only to death from the tumor per se [7]. Therefore, it is of vital importance to effectively control DVT, as it influences the vital prognosis. We recently encountered a patient with left internal jugular and subclavian vein thrombosis that developed during anticancer chemotherapy following surgery for ovarian cancer and report the case herein.

## 2. Case Presentation

The patient, a 47-year-old woman, visited our hospital with a 2-day history of a painful swelling on the left side of her neck, which was of sudden onset.

**Figure 1:**
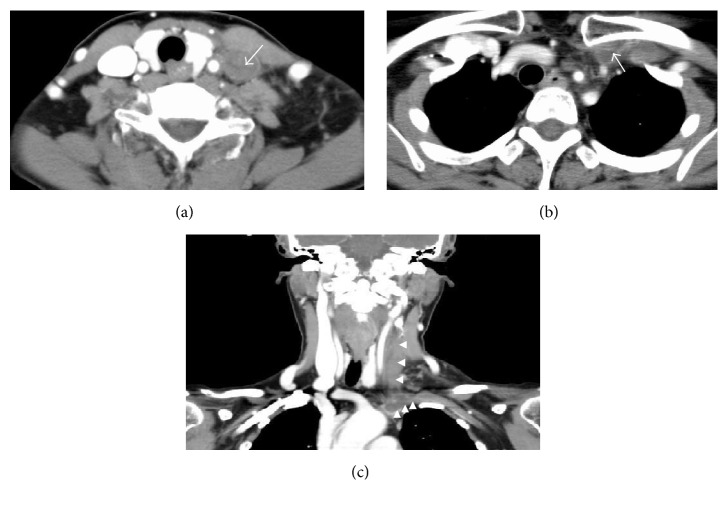
Axial enhanced CT image of soft tissue of the neck (a), the chest (b), and coronal enhanced CT image of soft tissue of the neck (c). There are filling defect in the left internal jugular vein (a) and subclavian vein (b), with the lumen filled with dense soft tissue (arrow). A vascular filling defect is noted in the region extending from the left internal jugular vein to the left subclavian vein, and the venous lumina are filled with dense soft tissue (arrowhead).

**Figure 2:**
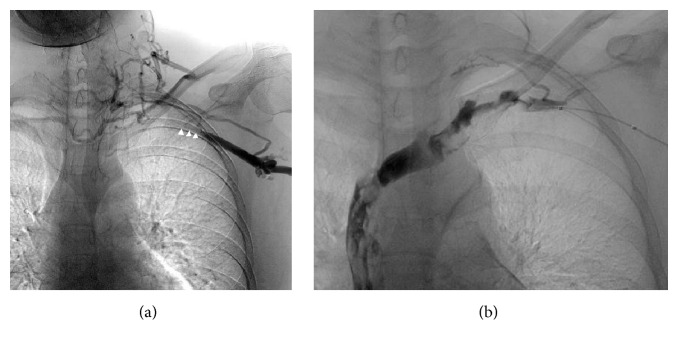
Left subclavian vein angiogram before (a) and after (b) dilation. Blood flow in the left subclavian vein to the left internal jugular vein is interrupted, while inflow of contrast medium is noted into the right subclavian vein via a collateral vessel (arrowhead in (a)). The left subclavian vein is visualized as a contrast-enhanced image after the balloon dilatation (b).

**Figure 3:**
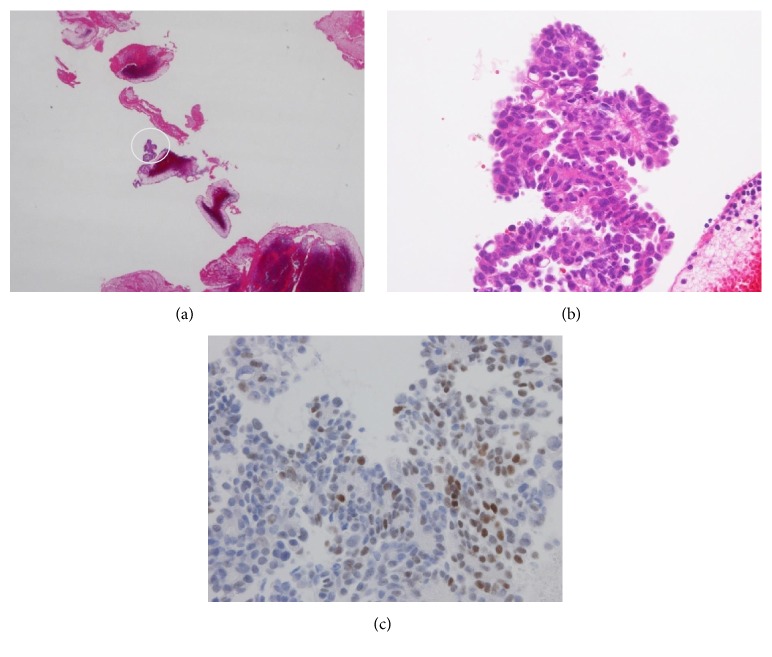
Histology of tumor cells in the blood clot (a)–(c). Tumor cells are seen in the blood clot (hematoxylin and eosin stain, ×100 (a)). Atypical cells with eosinophilic cytoplasm showing a high N/C ratio proliferating in a papillary pattern (hematoxylin and eosin stain, ×400 (b)). Note the positive (brownish) staining for estrogen receptors (Immunostaining for estrogen receptors (c)).

On initial examination, a swelling, tender to palpation, involving the region extending from the left neck to the shoulder was noted, with no redness of the overlying skin. The patient was receiving postoperative periodic chemotherapy following total hysterectomy with bilateral adnexectomy and omentectomy performed elsewhere 2 years earlier for ovarian cancer. The patient was not obese (body mass index: 22.3 kg/m^2^) and had no past history of central venous catheter insertion, ischemic disease, hypertension, diabetes, or hyperlipidemia. On initial examination, there were no abnormal findings in the oral cavity, and fiber-optic laryngoscopy did not reveal any evidence of inflammation, such as reddening or edema, nor was any tumor evident in the pharyngolaryngeal region. Hematologic examination showed the following: white blood cell count, 5,800/mm^3^; hemoglobin, 11.8 g/dL; platelet count, 234,000/mm^3^. The liver and kidney function test results were within normal limits, while the serum C-reactive protein was elevated to 4.51 mg/dL. Contrast-enhanced computed tomography (CT) of the neck, as part of the systematic medical workup, revealed a filling defect in the left internal jugular vein to left subclavian vein region, with the venous lumina filled with dense soft tissue. On the basis of the above findings, we diagnosed the patient as having thrombosis of the left internal jugular and left subclavian veins (Figures 1(a), 1(b), and 1(c)). With the diagnosis of UEDVT, blood coagulation tests were performed, which revealed that the prothrombin time, activated partial thromboplastin time, and antithrombin III levels were within normal limits, while the plasma d-dimer level was elevated to 1.5 g/mL (normal range: <1.0 g/mL). The patient was begun on treatment with oral rivaroxaban at the dose of 15 mg q.d. for the UEDVT on the 3rd hospital day. The left-sided neck pain improved with the treatment; however, the left shoulder pain became worse on the 19th hospital day, at which time the plasma d-dimer level was found to have further increased to 3.9 g/mL. On the 27th hospital day, catheter thrombectomy was performed because of failure of the rivaroxaban therapy to provide sufficient benefit. Removal of the thrombus via an 8 Fr catheter inserted through the left brachial vein and left internal jugular vein dilation with a 6 mm balloon resulted in a 50% reduction of the complete left subclavian vein occlusion. As elevation of the left upper extremity led to reocclusion of the left subclavian vein (even though the blood flow in the left upper limb remained intact when the limb was not elevated), an infusion catheter was placed in the vein for 2-day thrombolytic therapy with urokinase at 480,000 units/day. Angiographic examination on the 29th hospital day showed a collapsed left internal jugular vein and appearance of a collateral circulatory pathway. The thrombus in the left subclavian vein was found to have diminished in size, and while the blood flow in the left subclavian vein was interrupted upon elevation of the left upper extremity, the flow in the vein had improved to such an extent that the vessel became reperfused and unoccluded when the left upper limb was hung down (Figures 2(a) and 2(b)). Histopathologic examination of the removed thrombus revealed the presence of adenocarcinoma cells that showed positive staining for cytokeratin (AE1/AE3), ER, and PgR expressions, indicating hematogenous dissemination of malignant cells (Figures 3(a), 3(b), and 3(c)). The patient was then discharged from the hospital on oral rivaroxaban at 15 mg q.d.

## 3. Discussion

DVT occurs more frequently in the lower extremities and is unusual in the upper extremities, the latter accounting for only about 4% to 10% of all cases of DVT [1]. As for the etiology of secondary UEDVT, CVC is the most common cause (70%), while more than about 40% of cases of secondary UEDVT are reported to be cancer-related. The disorder is of unknown cause in 20% of the cases [8]. According to a systematic review by Bleker et al., secondary UEDVT is CVC-related in 53% of cases and cancer-related in 44%; hence, CVC and cancer, accounting for most of the cases, represent the most important predisposing factors for secondary UEDVT [1]. One large cohort study conducted previously showed that tumors of the bone, ovary, brain, and pancreas were associated with the highest risk of DVT [9]. Girolami et al. reported that idiopathic UEDVT was more frequently associated with occult cancers as compared to LEDVT and that lung cancer and lymphomas represented the majority of cancers associated with UEDVT [10].

Other factors also reported as independent risk factors for the development of DVT are as follows (in decreasing order of the odds ratio): surgery, trauma, hospital or nursing home confinement, malignancy, venous catheter or pacemaker insertion, superficial venous thrombosis, and neurological disease with extremity paresis [11].

Venous thrombosis is a paraneoplastic syndrome, where stasis of the regional blood flow is prone to occur because of direct tumor invasion or compression of the blood vessels by the tumor itself or by metastatic lymph nodes; in addition, hypercoagulability of the blood and vessel wall damage may also be involved. The hypercoagulable state may be attributed to the generation of clotting intermediates (e.g., tissue factor [TF], factor Xa, and thrombin), clotting or platelet function inhibitors (e.g., COX-2), or fibrinolysis inhibitors (e.g., plasminogen activator inhibitor, type 1 [PAI-1]) [12].

The risk of DVT is 4- to 7-fold greater in cases of malignancy and 6.5-fold higher in malignancy patients receiving chemotherapy as compared to that in those not receiving chemotherapy (4.1-fold higher) [9, 13]. The reported average incidence of recurrent DVT in cancer patients is 3.8% over a follow-up period of 3 to 13 months according to the results of a prospective study, with a 2- to 3-fold higher risk of recurrence in cancer patients as compared to noncancer patients [1, 14, 15]. In cancer patients, the average mortality rate is 18% at 3 months after the onset of UEDVT and 47% at 1 year after the onset. The risk of mortality at 3 months after the onset of UEDVT in cancer patients is reportedly 8 times as high as that in noncancer patients; thus DVT associated with cancer poses a life-threatening problem [1, 14].

Several excellent guidelines for the treatment of DVT have been published in recent years [16, 17]. It is recommended in the American College of Chest Physicians (ACCP) guideline that treatment of UEDVT be conducted on the basis of the treatment rationale for LEDVT, observational studies, and the results of studies on understanding of the natural history of UEDVT, since there have been no randomized controlled trials for the treatment of UEDVT [16]. The ACCP guideline recommends initial parenteral anticoagulant therapy or initial anticoagulation with rivaroxaban in patients with acute DVT, and we prescribed oral rivaroxaban in our present case documented herein [16]. It has been shown that single-drug therapy with rivaroxaban yields similar efficacy to that of standard therapy for DVT consisting of low-molecular-weight heparin (LMWH) combined with a vitamin K antagonist (VKA) and that the risk of major bleeding in patients receiving rivaroxaban therapy is significantly lower as compared to that in patients receiving LMWH [18]. Ageno et al. also reported that rivaroxaban is a safe and effective alternative to standard anticoagulant therapy and is highly advantageous in that it can be managed even at the outpatient service, whereas the standard therapy requires hospitalization for laboratory monitoring and dose adjustment [19]. In the present case, the left shoulder pain became worse on day 17 of the oral medication (19th hospital day), associated with elevation of the plasma d-dimer level despite the ongoing rivaroxaban therapy, suggestive of aggravation of the DVT. Because of the apparent failure of the rivaroxaban therapy to provide sufficient efficacy, we performed catheter thrombectomy and balloon angioplasty. On account of the potential difficulty in balloon angioplasty of the left internal jugular vein in this case, we conducted the procedure on the left subclavian vein, whereby a 50% reduction of the complete stenosis of the vein was achieved. However, mere elevation of the left upper extremity promptly resulted in reocclusion of the vessel, due in part to the rather extensive thrombosis. Since it is often the case that catheter thrombectomy and balloon angioplasty alone are insufficient to completely remove a venous thrombus, thrombolytic therapy was considered necessary as the next step of treatment in this case, and we decided to administer the thrombolytic therapy in consultation with the treating gynecologist. After placement of an infusion catheter, the patient was administered thrombolytic therapy for 2 days using urokinase, which resulted in reperfusion of the left subclavian vein, although angiography revealed interruption of the blood flow through the left internal jugular vein and inflow of contrast medium into the right subclavian vein via a collateral vessel. Pathologic examination of the removed thrombus revealed adenocarcinoma cells, suggestive of hematogenous dissemination from the ovarian cancer. Although needless to mention, it is ideal to accomplish radical cure of the primary disease in order to control the DVT; this was difficult to accomplish in the present case, because the patient already showed evidence of hematogenous tumor cell dissemination to the peritoneum. Therefore, as the risk of DVT recurrence is expected to persist, we propose to manage the patient with further continuation of the anticoagulant therapy as well as chemotherapy.

## 4. Conclusion

We recently encountered a case of UEDVT in a patient who presented to our outpatient service with the chief complaints of left-sided neck pain and swelling. There are a variety of disorders that can produce neck swelling, including inflammation and tumor. UEDVT is a rare disorder among outpatients without an inserted CVC, and its incidence rate is much lower (4% to 10%) as compared to that of LEDVT. It would presumably be difficult to promptly make a diagnostic differentiation of UEDVT from other disorders. However, the diagnosis of UEDVT should not be overlooked, as it may give rise to serious complications such as PE, and thus have a great impact on the vital prognosis. We thus recommend that otolaryngologists gain enough understanding of the close relationship between CVC insertion and cancer as risk factors for DVT. They must obtain a detailed history and perform careful clinical examination, keeping in mind the possibility of UEDVT, in cases with a CVC or cancer.
